# Supplementary material for: Changes in Health Care Access during the COVID-19 Pandemic: Estimates of National Japanese Data, June 2020–October 2021
Source: Int J Environ Res Public Health. 2022 Jul 20;19(14):8810. doi: 10.3390/ijerph19148810 (PMC9317863; doi:10.3390/ijerph19148810)
Supplement: Supplementary file 1 [file ijerph-19-08810-s001.zip › ijerph-1777547-supplementary.pdf]

# Online Supplementary Material.

**Table of Contents**


| Table/Figure | Title | Page |
|---|---|---|
| Table S1 | Monthly average number of outpatients per day at hospitals in Japan, June 2020-October 2021. | 2-3 |
| Table S2 | Monthly average number of inpatients per day in Japan, June 2020-October 2021. | 4-5 |
| Table S3 | Monthly average length of hospital stay per patient in Japan, June 2020-October 2021. | 6-7 |
| Table S4 | Monthly number of new hospitalizations in Japan, June 2020-October 2021. | 8-10 |

## Table S1. Monthly average number of outpatients per day at hospitals in Japan, January 2020-October 2021.

| Hospital Type | Month-Year | Observed | Excess | % Excess | Exiguous | % Exiguous |
|---|---|---|---|---|---|---|
| General hospital | | | | | | |
| | Jun-20 | 1145535 | 0-0 | 0.00-0.00 | 25704-110786 | 2.05-8.82 |
| | Jul-20 | 1160820 | 0-0 | 0.00-0.00 | 31360-108344 | 2.47-8.54 |
| | Aug-20 | 1101002 | 0-0 | 0.00-0.00 | 73470-149900 | 5.87-11.98 |
| | Sep-20 | 1172387 | 0-0 | 0.00-0.00 | 9202-88527 | 0.73-7.02 |
| | Oct-20 | 1245515 | 0-0 | 0.00-0.00 | 0-20475 | 0.00-1.62 |
| | Nov-20 | 1165447 | 0-0 | 0.00-0.00 | 29549-107275 | 2.32-8.43 |
| | Dec-20 | 1173032 | 0-0 | 0.00-0.00 | 0-76114 | 0.00-6.09 |
| | Jan-21 | 1045311 | 0-0 | 0.00-0.00 | 114863-191226 | 9.29-15.46 |
| | Feb-21 | 1123639 | 0-0 | 0.00-0.00 | 43561-122183 | 3.50-9.81 |
| | Mar-21 | 1234309 | 0-0 | 0.00-0.00 | 0-26042 | 0.00-2.07 |
| | Apr-21 | 1197703 | 0-0 | 0.00-0.00 | 0-36056 | 0.00-2.92 |
| | May-21 | 1062361 | 0-0 | 0.00-0.00 | 105198-176846 | 8.49-14.27 |
| | Jun-21 | 1238764 | 0-0 | 0.00-0.00 | 0-12238 | 0.00-0.98 |
| | Jul-21 | 1190787 | 0-0 | 0.00-0.00 | 1597-70266 | 0.13-5.57 |
| | Aug-21 | 1197168 | 0-0 | 0.00-0.00 | 0-47924 | 0.00-3.85 |
| | Sep-21 | 1223731 | 0-0 | 0.00-0.00 | 0-26840 | 0.00-2.15 |
| | Oct-21 | 1217300 | 0-0 | 0.00-0.00 | 0-41034 | 0.00-3.26 |
| Psychiatric hospital | | | | | | |
| | Jun-20 | 56883 | 0-0 | 0.00-0.00 | 0-2329 | 0.00-3.93 |
| | Jul-20 | 56814 | 0-0 | 0.00-0.00 | 0-2869 | 0.00-4.81 |
| | Aug-20 | 53453 | 0-0 | 0.00-0.00 | 1725-5650 | 2.92-9.56 |
| | Sep-20 | 56689 | 0-0 | 0.00-0.00 | 0-2849 | 0.00-4.78 |
| | Oct-20 | 59519 | 0-0 | 0.00-0.00 | 0-22 | 0.00-0.04 |
| | Nov-20 | 55226 | 0-0 | 0.00-0.00 | 247-4221 | 0.42-7.10 |
| | Dec-20 | 56776 | 0-0 | 0.00-0.00 | 0-1229 | 0.00-2.12 |
| | Jan-21 | 50781 | 0-0 | 0.00-0.00 | 3019-6628 | 5.26-11.55 |
| | Feb-21 | 55416 | 0-0 | 0.00-0.00 | 0-2680 | 0.00-4.61 |
| | Mar-21 | 59728 | 0-0 | 0.00-0.00 | 0-339 | 0.00-0.56 |
| | Apr-21 | 59122 | 171-0 | 0.29-0.00 | 0-0 | 0.00-0.00 |
| | May-21 | 51584 | 0-0 | 0.00-0.00 | 3730-7506 | 6.31-12.70 |
| | Jun-21 | 58698 | 0-0 | 0.00-0.00 | 0-624 | 0.00-1.05 |
| | Jul-21 | 57202 | 0-0 | 0.00-0.00 | 0-2338 | 0.00-3.93 |
| | Aug-21 | 55546 | 0-0 | 0.00-0.00 | 0-3374 | 0.00-5.73 |
| | Sep-21 | 58021 | 0-0 | 0.00-0.00 | 0-1183 | 0.00-2.00 |
| | Oct-21 | 58075 | 0-0 | 0.00-0.00 | 0-1224 | 0.00-2.06 |
| Total | | | | | | |
| | Jun-20 | 1202418 | 0-0 | 0.00-0.00 | 24355-113081 | 1.85-8.60 |
| | Jul-20 | 1217634 | 0-0 | 0.00-0.00 | 30758-111182 | 2.31-8.37 |
| | Aug-20 | 1154455 | 0-0 | 0.00-0.00 | 75372-155512 | 5.75-11.87 |
| | Sep-20 | 1229076 | 0-0 | 0.00-0.00 | 8562-91338 | 0.65-6.92 |
| | Oct-20 | 1305034 | 0-0 | 0.00-0.00 | 0-20468 | 0.00-1.54 |
| | Nov-20 | 1220673 | 0-0 | 0.00-0.00 | 29994-111482 | 2.25-8.37 |

| | | | | | | |
|---|---|---|---|---|---|---|
| | Dec-20 | 1229808 | 0-0 | 0.00-0.00 | 0-77342 | 0.00-5.92 |
| | Jan-21 | 1096091 | 0-0 | 0.00-0.00 | 118082-197856 | 9.13-15.29 |
| | Feb-21 | 1179055 | 0-0 | 0.00-0.00 | 42451-124858 | 3.26-9.58 |
| | Mar-21 | 1294037 | 0-0 | 0.00-0.00 | 0-26330 | 0.00-1.99 |
| | Apr-21 | 1256825 | 0-0 | 0.00-0.00 | 0-35833 | 0.00-2.77 |
| | May-21 | 1113945 | 0-0 | 0.00-0.00 | 109115-184306 | 8.40-14.20 |
| | Jun-21 | 1297463 | 0-0 | 0.00-0.00 | 0-12823 | 0.00-0.98 |
| | Jul-21 | 1247989 | 0-0 | 0.00-0.00 | 729-72575 | 0.06-5.50 |
| | Aug-21 | 1252714 | 0-0 | 0.00-0.00 | 0-51266 | 0.00-3.93 |
| | Sep-21 | 1281753 | 0-0 | 0.00-0.00 | 0-27988 | 0.00-2.14 |
| | Oct-21 | 1275375 | 0-0 | 0.00-0.00 | 0-42235 | 0.00-3.21 |

**Table S2. Monthly average number of inpatients per day in Japan, January 2020-October 2021.**

| Bed Type | Month-Year | Observed | Excess | % Excess | Exiguous | % Exiguous |
|---|---|---|---|---|---|---|
| General beds | | | | | | |
| | Jun-20 | 606442 | 0-0 | 0.00-0.00 | 53019-69453 | 7.84-10.28 |
| | Jul-20 | 624200 | 0-0 | 0.00-0.00 | 35418-56275 | 5.20-8.27 |
| | Aug-20 | 627843 | 0-0 | 0.00-0.00 | 37967-52433 | 5.58-7.71 |
| | Sep-20 | 629621 | 0-0 | 0.00-0.00 | 31584-48255 | 4.66-7.12 |
| | Oct-20 | 636628 | 0-0 | 0.00-0.00 | 19795-41271 | 2.92-6.09 |
| | Nov-20 | 638290 | 0-0 | 0.00-0.00 | 27348-41984 | 4.02-6.17 |
| | Dec-20 | 625235 | 0-0 | 0.00-0.00 | 42143-59228 | 6.16-8.65 |
| | Jan-21 | 626119 | 0-0 | 0.00-0.00 | 43607-65879 | 6.30-9.52 |
| | Feb-21 | 636429 | 0-0 | 0.00-0.00 | 51159-66486 | 7.28-9.46 |
| | Mar-21 | 633087 | 0-0 | 0.00-0.00 | 47933-65643 | 6.86-9.39 |
| | Apr-21 | 625590 | 0-0 | 0.00-0.00 | 37590-58863 | 5.49-8.60 |
| | May-21 | 603064 | 0-0 | 0.00-0.00 | 61494-75780 | 9.06-11.16 |
| | Jun-21 | 608020 | 0-0 | 0.00-0.00 | 52565-68997 | 7.76-10.19 |
| | Jul-21 | 613338 | 0-0 | 0.00-0.00 | 47379-67790 | 6.96-9.95 |
| | Aug-21 | 617473 | 0-0 | 0.00-0.00 | 50032-64129 | 7.34-9.41 |
| | Sep-21 | 604369 | 0-0 | 0.00-0.00 | 56899-73325 | 8.40-10.82 |
| | Oct-21 | 611561 | 0-0 | 0.00-0.00 | 44104-65161 | 6.52-9.63 |
| Long-term care beds covered by long-term care insurance (LTCI) | | | | | | |
| | Jun-20 | 15398 | 0-0 | 0.00-0.00 | 9583-13087 | 33.64-45.94 |
| | Jul-20 | 15075 | 0-0 | 0.00-0.00 | 9175-12730 | 33.00-45.78 |
| | Aug-20 | 14955 | 0-0 | 0.00-0.00 | 8962-12416 | 32.74-45.36 |
| | Sep-20 | 14398 | 0-0 | 0.00-0.00 | 9001-12383 | 33.61-46.24 |
| | Oct-20 | 13841 | 0-0 | 0.00-0.00 | 9035-12327 | 34.53-47.11 |
| | Nov-20 | 13624 | 0-0 | 0.00-0.00 | 8799-11974 | 34.37-46.78 |
| | Dec-20 | 13445 | 0-0 | 0.00-0.00 | 8602-11724 | 34.18-46.58 |
| | Jan-21 | 13165 | 0-0 | 0.00-0.00 | 8613-11680 | 34.67-47.01 |
| | Feb-21 | 13060 | 0-0 | 0.00-0.00 | 8520-11506 | 34.68-46.84 |
| | Mar-21 | 12906 | 0-0 | 0.00-0.00 | 8141-11015 | 34.03-46.05 |
| | Apr-21 | 12460 | 0-0 | 0.00-0.00 | 8090-10836 | 34.73-46.51 |
| | May-21 | 12144 | 0-0 | 0.00-0.00 | 7995-10580 | 35.18-46.56 |
| | Jun-21 | 11674 | 0-0 | 0.00-0.00 | 8057-10598 | 36.18-47.58 |
| | Jul-21 | 11148 | 0-0 | 0.00-0.00 | 8195-10658 | 37.58-48.88 |
| | Aug-21 | 10711 | 0-0 | 0.00-0.00 | 8317-10675 | 38.89-49.92 |
| | Sep-21 | 10466 | 0-0 | 0.00-0.00 | 8016-10325 | 38.56-49.66 |
| | Oct-21 | 8903 | 0-0 | 0.00-0.00 | 9053-11292 | 44.83-55.91 |
| Tuberculosis care beds | | | | | | |
| | Jun-20 | 1279 | 0-0 | 0.00-0.00 | 49-128 | 3.48-9.07 |
| | Jul-20 | 1404 | 0-0 | 0.00-0.00 | 0-10 | 0.00-0.69 |
| | Aug-20 | 1424 | 11-0 | 0.80-0.00 | 0-0 | 0.00-0.00 |
| | Sep-20 | 1337 | 0-0 | 0.00-0.00 | 0-68 | 0.00-4.84 |
| | Oct-20 | 1330 | 0-0 | 0.00-0.00 | 0-58 | 0.00-4.18 |
| | Nov-20 | 1306 | 0-0 | 0.00-0.00 | 0-51 | 0.00-3.78 |
| | Dec-20 | 1284 | 0-0 | 0.00-0.00 | 0-39 | 0.00-2.92 |
| | Jan-21 | 1203 | 0-0 | 0.00-0.00 | 9-82 | 0.70-6.35 |
| | Feb-21 | 1117 | 0-0 | 0.00-0.00 | 71-140 | 5.65-11.15 |
| | Mar-21 | 1124 | 0-0 | 0.00-0.00 | 59-133 | 4.69-10.60 |
| | Apr-21 | 1156 | 0-0 | 0.00-0.00 | 34-103 | 2.70-8.18 |
| | May-21 | 1178 | 0-0 | 0.00-0.00 | 18-87 | 1.42-6.88 |
| | Jun-21 | 1117 | 0-0 | 0.00-0.00 | 87-161 | 6.81-12.58 |
| | Jul-21 | 1136 | 0-0 | 0.00-0.00 | 81-151 | 6.30-11.71 |
| | Aug-21 | 1204 | 0-0 | 0.00-0.00 | 10-80 | 0.78-6.25 |
| | Sep-21 | 1194 | 0-0 | 0.00-0.00 | 7-82 | 0.55-6.44 |
| | Oct-21 | 1140 | 0-0 | 0.00-0.00 | 45-113 | 3.59-9.05 |

| | | | | | | |
|---|---|---|---|---|---|---|
| Psychiatric care beds | | | | | | |
| | Jun-20 | 275436 | 0-0 | 0.00-0.00 | 2871-4412 | 1.03-1.58 |
| | Jul-20 | 277282 | 0-0 | 0.00-0.00 | 1632-3240 | 0.58-1.15 |
| | Aug-20 | 277321 | 0-0 | 0.00-0.00 | 2123-3444 | 0.76-1.23 |
| | Sep-20 | 276805 | 0-0 | 0.00-0.00 | 1769-3333 | 0.63-1.19 |
| | Oct-20 | 275617 | 0-0 | 0.00-0.00 | 1689-3326 | 0.61-1.19 |
| | Nov-20 | 274223 | 0-0 | 0.00-0.00 | 2179-3523 | 0.78-1.27 |
| | Dec-20 | 272587 | 0-0 | 0.00-0.00 | 2972-4470 | 1.07-1.61 |
| | Jan-21 | 271247 | 0-0 | 0.00-0.00 | 4104-5678 | 1.48-2.05 |
| | Feb-21 | 271358 | 0-0 | 0.00-0.00 | 4456-5802 | 1.61-2.09 |
| | Mar-21 | 270929 | 0-0 | 0.00-0.00 | 4473-6006 | 1.62-2.17 |
| | Apr-21 | 270615 | 0-0 | 0.00-0.00 | 4236-5831 | 1.53-2.11 |
| | May-21 | 269794 | 0-0 | 0.00-0.00 | 5431-6787 | 1.96-2.45 |
| | Jun-21 | 271014 | 0-0 | 0.00-0.00 | 4504-6074 | 1.63-2.19 |
| | Jul-21 | 272190 | 0-0 | 0.00-0.00 | 3929-5572 | 1.41-2.01 |
| | Aug-21 | 271615 | 0-0 | 0.00-0.00 | 5024-6379 | 1.81-2.29 |
| | Sep-21 | 270607 | 0-0 | 0.00-0.00 | 5176-6708 | 1.87-2.42 |
| | Oct-21 | 269994 | 0-0 | 0.00-0.00 | 4482-6091 | 1.62-2.21 |
| Total | | | | | | |
| | Jun-20 | 1132251 | 0-0 | 0.00-0.00 | 74068-93461 | 6.04-7.63 |
| | Jul-20 | 1152518 | 0-0 | 0.00-0.00 | 53550-77704 | 4.35-6.32 |
| | Aug-20 | 1157587 | 0-0 | 0.00-0.00 | 54787-72038 | 4.46-5.86 |
| | Sep-20 | 1159073 | 0-0 | 0.00-0.00 | 46199-66017 | 3.77-5.39 |
| | Oct-20 | 1162959 | 0-0 | 0.00-0.00 | 34461-59230 | 2.82-4.85 |
| | Nov-20 | 1164670 | 0-0 | 0.00-0.00 | 40602-57911 | 3.32-4.74 |
| | Dec-20 | 1152660 | 0-0 | 0.00-0.00 | 53108-73186 | 4.33-5.97 |
| | Jan-21 | 1155071 | 0-0 | 0.00-0.00 | 52788-78261 | 4.28-6.35 |
| | Feb-21 | 1164661 | 0-0 | 0.00-0.00 | 62672-80590 | 5.03-6.47 |
| | Mar-21 | 1157974 | 0-0 | 0.00-0.00 | 61785-82260 | 4.98-6.63 |
| | Apr-21 | 1151297 | 0-0 | 0.00-0.00 | 48544-72874 | 3.97-5.95 |
| | May-21 | 1128836 | 0-0 | 0.00-0.00 | 71665-88342 | 5.89-7.26 |
| | Jun-21 | 1131535 | 0-0 | 0.00-0.00 | 63471-82823 | 5.23-6.82 |
| | Jul-21 | 1136629 | 0-0 | 0.00-0.00 | 58628-81808 | 4.81-6.71 |
| | Aug-21 | 1148034 | 0-0 | 0.00-0.00 | 54170-70262 | 4.45-5.77 |
| | Sep-21 | 1130001 | 0-0 | 0.00-0.00 | 62945-81818 | 5.19-6.75 |
| | Oct-21 | 1126324 | 0-0 | 0.00-0.00 | 57800-81274 | 4.79-6.73 |
| Long-term care beds | | | | | | |
| | Jun-20 | 248918 | 0-0 | 0.00-0.00 | 14258-19864 | 5.30-7.39 |
| | Jul-20 | 249018 | 0-0 | 0.00-0.00 | 13180-19120 | 4.92-7.13 |
| | Aug-20 | 248646 | 0-0 | 0.00-0.00 | 13310-18847 | 4.98-7.05 |
| | Sep-20 | 249234 | 0-0 | 0.00-0.00 | 11201-16743 | 4.21-6.29 |
| | Oct-20 | 247453 | 0-0 | 0.00-0.00 | 11175-16930 | 4.23-6.40 |
| | Nov-20 | 247205 | 0-0 | 0.00-0.00 | 11184-16509 | 4.24-6.26 |
| | Dec-20 | 247257 | 0-0 | 0.00-0.00 | 11039-16355 | 4.19-6.20 |
| | Jan-21 | 247389 | 0-0 | 0.00-0.00 | 11569-17177 | 4.37-6.49 |
| | Feb-21 | 249197 | 0-0 | 0.00-0.00 | 11153-16345 | 4.20-6.16 |
| | Mar-21 | 248496 | 0-0 | 0.00-0.00 | 11155-16268 | 4.21-6.14 |
| | Apr-21 | 247160 | 0-0 | 0.00-0.00 | 10126-15416 | 3.86-5.87 |
| | May-21 | 244330 | 0-0 | 0.00-0.00 | 11899-16691 | 4.56-6.39 |
| | Jun-21 | 245106 | 0-0 | 0.00-0.00 | 9588-14384 | 3.69-5.54 |
| | Jul-21 | 244510 | 0-0 | 0.00-0.00 | 9390-14343 | 3.63-5.54 |
| | Aug-21 | 244251 | 0-0 | 0.00-0.00 | 9378-13848 | 3.63-5.37 |
| | Sep-21 | 243861 | 0-0 | 0.00-0.00 | 7892-12317 | 3.08-4.81 |
| | Oct-21 | 241535 | 0-0 | 0.00-0.00 | 8100-12700 | 3.19-5.00 |

**Table S3. Monthly average length of hospital stay per patient in Japan, January 2020-October 2021.**

| Bed Type | Month-Year | Observed | Excess | % Excess | Exiguous | % Exiguous |
|---|---|---|---|---|---|---|
| General beds | | | | | | |
| | Jun-20 | 16 | 0-0 | 0.61-0.00 | 0-0 | 0.00-0.00 |
| | Jul-20 | 16 | 0-0 | 1.16-0.00 | 0-0 | 0.00-0.00 |
| | Aug-20 | 16 | 0-0 | 1.10-0.00 | 0-0 | 0.00-0.00 |
| | Sep-20 | 16 | 0-0 | 0.84-0.00 | 0-0 | 0.00-0.00 |
| | Oct-20 | 16 | 0-0 | 0.76-0.00 | 0-0 | 0.00-0.00 |
| | Nov-20 | 16 | 0-0 | 0.68-0.00 | 0-0 | 0.00-0.00 |
| | Dec-20 | 16 | 0-0 | 0.00-0.00 | 0-0 | 0.00-0.71 |
| | Jan-21 | 18 | 2-0 | 10.99-0.00 | 0-0 | 0.00-0.00 |
| | Feb-21 | 17 | 1-0 | 4.59-0.00 | 0-0 | 0.00-0.00 |
| | Mar-21 | 16 | 0-0 | 0.84-0.00 | 0-0 | 0.00-0.00 |
| | Apr-21 | 16 | 0-0 | 0.92-0.00 | 0-0 | 0.00-0.00 |
| | May-21 | 17 | 1-0 | 7.14-0.00 | 0-0 | 0.00-0.00 |
| | Jun-21 | 16 | 0-0 | 1.85-0.00 | 0-0 | 0.00-0.00 |
| | Jul-21 | 16 | 0-0 | 1.58-0.00 | 0-0 | 0.00-0.00 |
| | Aug-21 | 16 | 0-0 | 1.39-0.00 | 0-0 | 0.00-0.00 |
| | Sep-21 | 16 | 0-0 | 1.10-0.00 | 0-0 | 0.00-0.00 |
| | Oct-21 | 16 | 0-0 | 1.01-0.00 | 0-0 | 0.00-0.00 |
| Long-term care beds covered by long-term care insurance (LTCI) | | | | | | |
| | Jun-20 | 305 | 0-0 | 0.00-0.00 | 0-8 | 0.00-2.53 |
| | Jul-20 | 335 | 20-0 | 6.38-0.00 | 0-0 | 0.00-0.00 |
| | Aug-20 | 319 | 2-0 | 0.71-0.00 | 0-0 | 0.00-0.00 |
| | Sep-20 | 315 | 4-0 | 1.22-0.00 | 0-0 | 0.00-0.00 |
| | Oct-20 | 346 | 39-4 | 12.78-1.30 | 0-0 | 0.00-0.00 |
| | Nov-20 | 341 | 37-2 | 12.29-0.66 | 0-0 | 0.00-0.00 |
| | Dec-20 | 347 | 40-4 | 12.97-1.30 | 0-0 | 0.00-0.00 |
| | Jan-21 | 355 | 48-13 | 15.79-4.24 | 0-0 | 0.00-0.00 |
| | Feb-21 | 357 | 57-21 | 18.81-6.99 | 0-0 | 0.00-0.00 |
| | Mar-21 | 330 | 42-8 | 14.69-2.78 | 0-0 | 0.00-0.00 |
| | Apr-21 | 334 | 35-0 | 11.77-0.00 | 0-0 | 0.00-0.00 |
| | May-21 | 385 | 76-40 | 24.72-12.96 | 0-0 | 0.00-0.00 |
| | Jun-21 | 312 | 0-0 | 0.00-0.00 | 0-8 | 0.00-2.38 |
| | Jul-21 | 334 | 12-0 | 3.85-0.00 | 0-0 | 0.00-0.00 |
| | Aug-21 | 351 | 30-0 | 9.26-0.00 | 0-0 | 0.00-0.00 |
| | Sep-21 | 264 | 0-0 | 0.00-0.00 | 21-55 | 6.58-17.22 |
| | Oct-21 | 288 | 0-0 | 0.00-0.00 | 0-26 | 0.00-8.20 |
| Tuberculosis care beds | | | | | | |
| | Jun-20 | 50 | 0-0 | 0.00-0.00 | 0-15 | 0.00-23.06 |
| | Jul-20 | 42 | 0-0 | 0.00-0.00 | 7-22 | 10.93-34.44 |
| | Aug-20 | 48 | 0-0 | 0.00-0.00 | 2-18 | 3.05-26.80 |
| | Sep-20 | 57 | 0-0 | 0.00-0.00 | 0-8 | 0.00-12.39 |
| | Oct-20 | 59 | 0-0 | 0.00-0.00 | 0-6 | 0.00-9.33 |
| | Nov-20 | 57 | 0-0 | 0.00-0.00 | 0-6 | 0.00-9.47 |
| | Dec-20 | 47 | 0-0 | 0.00-0.00 | 1-16 | 1.58-25.70 |
| | Jan-21 | 50 | 0-0 | 0.00-0.00 | 0-13 | 0.00-20.14 |
| | Feb-21 | 56 | 0-0 | 0.00-0.00 | 0-7 | 0.00-10.96 |
| | Mar-21 | 51 | 0-0 | 0.00-0.00 | 0-11 | 0.00-17.80 |
| | Apr-21 | 49 | 0-0 | 0.00-0.00 | 0-15 | 0.00-23.24 |
| | May-21 | 48 | 0-0 | 0.00-0.00 | 1-16 | 1.56-25.28 |
| | Jun-21 | 53 | 0-0 | 0.00-0.00 | 0-12 | 0.00-18.10 |
| | Jul-21 | 48 | 0-0 | 0.00-0.00 | 1-16 | 1.57-24.60 |
| | Aug-21 | 42 | 0-0 | 0.00-0.00 | 8-23 | 12.33-35.25 |
| | Sep-21 | 48 | 0-0 | 0.00-0.00 | 2-16 | 3.10-25.51 |
| | Oct-21 | 59 | 0-0 | 0.00-0.00 | 0-5 | 0.00-7.78 |

| | | | | | | |
|---|---|---|---|---|---|---|
| Psychiatric care beds | | | | | | |
| | Jun-20 | 265 | 10-0 | 3.87-0.00 | 0-0 | 0.00-0.00 |
| | Jul-20 | 270 | 15-0 | 5.82-0.00 | 0-0 | 0.00-0.00 |
| | Aug-20 | 279 | 20-0 | 7.53-0.00 | 0-0 | 0.00-0.00 |
| | Sep-20 | 268 | 8-0 | 3.14-0.00 | 0-0 | 0.00-0.00 |
| | Oct-20 | 258 | 0-0 | 0.00-0.00 | 0-4 | 0.00-1.49 |
| | Nov-20 | 279 | 17-0 | 6.46-0.00 | 0-0 | 0.00-0.00 |
| | Dec-20 | 271 | 3-0 | 1.01-0.00 | 0-0 | 0.00-0.00 |
| | Jan-21 | 308 | 38-5 | 14.21-1.85 | 0-0 | 0.00-0.00 |
| | Feb-21 | 281 | 14-0 | 5.38-0.00 | 0-0 | 0.00-0.00 |
| | Mar-21 | 259 | 0-0 | 0.19-0.00 | 0-0 | 0.00-0.00 |
| | Apr-21 | 264 | 6-0 | 2.18-0.00 | 0-0 | 0.00-0.00 |
| | May-21 | 298 | 41-9 | 16.09-3.51 | 0-0 | 0.00-0.00 |
| | Jun-21 | 265 | 11-0 | 4.22-0.00 | 0-0 | 0.00-0.00 |
| | Jul-21 | 273 | 18-0 | 6.88-0.00 | 0-0 | 0.00-0.00 |
| | Aug-21 | 278 | 19-0 | 7.32-0.00 | 0-0 | 0.00-0.00 |
| | Sep-21 | 272 | 12-0 | 4.58-0.00 | 0-0 | 0.00-0.00 |
| | Oct-21 | 273 | 12-0 | 4.56-0.00 | 0-0 | 0.00-0.00 |
| Total | | | | | | |
| | Jun-20 | 28 | 1-0 | 4.26-0.00 | 0-0 | 0.00-0.00 |
| | Jul-20 | 28 | 2-0 | 6.24-0.00 | 0-0 | 0.00-0.00 |
| | Aug-20 | 28 | 1-0 | 4.90-0.00 | 0-0 | 0.00-0.00 |
| | Sep-20 | 28 | 1-0 | 5.01-0.00 | 0-0 | 0.00-0.00 |
| | Oct-20 | 27 | 0-0 | 0.76-0.00 | 0-0 | 0.00-0.00 |
| | Nov-20 | 28 | 2-0 | 6.19-0.00 | 0-0 | 0.00-0.00 |
| | Dec-20 | 27 | 0-0 | 0.88-0.00 | 0-0 | 0.00-0.00 |
| | Jan-21 | 30 | 3-0 | 11.78-0.00 | 0-0 | 0.00-0.00 |
| | Feb-21 | 29 | 2-0 | 6.68-0.00 | 0-0 | 0.00-0.00 |
| | Mar-21 | 27 | 0-0 | 1.64-0.00 | 0-0 | 0.00-0.00 |
| | Apr-21 | 27 | 0-0 | 0.97-0.00 | 0-0 | 0.00-0.00 |
| | May-21 | 29 | 2-0 | 9.07-0.00 | 0-0 | 0.00-0.00 |
| | Jun-21 | 27 | 1-0 | 3.25-0.00 | 0-0 | 0.00-0.00 |
| | Jul-21 | 27 | 1-0 | 4.78-0.00 | 0-0 | 0.00-0.00 |
| | Aug-21 | 27 | 1-0 | 3.11-0.00 | 0-0 | 0.00-0.00 |
| | Sep-21 | 27 | 1-0 | 3.34-0.00 | 0-0 | 0.00-0.00 |
| | Oct-21 | 27 | 1-0 | 2.95-0.00 | 0-0 | 0.00-0.00 |
| Long-term care beds | | | | | | |
| | Jun-20 | 135 | 2-0 | 1.25-0.00 | 0-0 | 0.00-0.00 |
| | Jul-20 | 140 | 7-0 | 5.62-0.00 | 0-0 | 0.00-0.00 |
| | Aug-20 | 140 | 6-0 | 4.81-0.00 | 0-0 | 0.00-0.00 |
| | Sep-20 | 135 | 3-0 | 2.36-0.00 | 0-0 | 0.00-0.00 |
| | Oct-20 | 131 | 1-0 | 0.41-0.00 | 0-0 | 0.00-0.00 |
| | Nov-20 | 134 | 7-0 | 5.52-0.00 | 0-0 | 0.00-0.00 |
| | Dec-20 | 124 | 0-0 | 0.00-0.00 | 0-3 | 0.00-2.68 |
| | Jan-21 | 141 | 15-0 | 11.69-0.00 | 0-0 | 0.00-0.00 |
| | Feb-21 | 131 | 5-0 | 3.80-0.00 | 0-0 | 0.00-0.00 |
| | Mar-21 | 126 | 3-0 | 2.11-0.00 | 0-0 | 0.00-0.00 |
| | Apr-21 | 128 | 2-0 | 1.56-0.00 | 0-0 | 0.00-0.00 |
| | May-21 | 145 | 18-0 | 13.89-0.00 | 0-0 | 0.00-0.00 |
| | Jun-21 | 132 | 3-0 | 2.58-0.00 | 0-0 | 0.00-0.00 |
| | Jul-21 | 137 | 8-0 | 6.56-0.00 | 0-0 | 0.00-0.00 |
| | Aug-21 | 133 | 4-0 | 2.82-0.00 | 0-0 | 0.00-0.00 |
| | Sep-21 | 131 | 3-0 | 2.14-0.00 | 0-0 | 0.00-0.00 |
| | Oct-21 | 129 | 3-0 | 2.05-0.00 | 0-0 | 0.00-0.00 |

**Table S4. Monthly number of new hospitalizations in Japan, January 2020-October 2021.**

| Bed Type | Month-Year | Observed | Excess | % Excess | Exiguous | % Exiguous |
|---|---|---|---|---|---|---|
| General beds | | | | | | |
| | Jun-20 | 1158101 | 0-0 | 0.00-0.00 | 81683-170177 | 6.15-12.81 |
| | Jul-20 | 1215062 | 0-0 | 0.00-0.00 | 62283-139607 | 4.60-10.31 |
| | Aug-20 | 1218319 | 0-0 | 0.00-0.00 | 32076-117858 | 2.40-8.82 |
| | Sep-20 | 1182529 | 0-0 | 0.00-0.00 | 63611-147685 | 4.78-11.10 |
| | Oct-20 | 1260436 | 0-0 | 0.00-0.00 | 0-48566 | 0.00-3.71 |
| | Nov-20 | 1190471 | 0-0 | 0.00-0.00 | 42545-127614 | 3.23-9.68 |
| | Dec-20 | 1176219 | 0-0 | 0.00-0.00 | 56032-140342 | 4.26-10.66 |
| | Jan-21 | 1148090 | 0-0 | 0.00-0.00 | 74286-153585 | 5.71-11.80 |
| | Feb-21 | 1044546 | 0-0 | 0.00-0.00 | 182681-269879 | 13.90-20.53 |
| | Mar-21 | 1250967 | 0-0 | 0.00-0.00 | 0-38557 | 0.00-2.99 |
| | Apr-21 | 1175043 | 0-0 | 0.00-0.00 | 57117-138645 | 4.35-10.55 |
| | May-21 | 1101187 | 0-0 | 0.00-0.00 | 119906-207879 | 9.16-15.88 |
| | Jun-21 | 1176133 | 0-0 | 0.00-0.00 | 80683-166837 | 6.01-12.42 |
| | Jul-21 | 1201151 | 0-0 | 0.00-0.00 | 86158-168290 | 6.29-12.29 |
| | Aug-21 | 1223462 | 0-0 | 0.00-0.00 | 36559-127414 | 2.71-9.43 |
| | Sep-21 | 1134080 | 0-0 | 0.00-0.00 | 118498-205711 | 8.84-15.35 |
| | Oct-21 | 1211776 | 0-0 | 0.00-0.00 | 29395-111930 | 2.22-8.46 |
| Long-term care beds covered by long-term care insurance (LTCI) | | | | | | |
| | Jun-20 | 783 | 0-0 | 0.00-0.00 | 637-945 | 36.87-54.68 |
| | Jul-20 | 795 | 0-0 | 0.00-0.00 | 590-899 | 34.83-53.07 |
| | Aug-20 | 733 | 0-0 | 0.00-0.00 | 591-898 | 36.24-55.05 |
| | Sep-20 | 730 | 0-0 | 0.00-0.00 | 581-878 | 36.12-54.61 |
| | Oct-20 | 749 | 0-0 | 0.00-0.00 | 524-812 | 33.57-52.02 |
| | Nov-20 | 642 | 0-0 | 0.00-0.00 | 636-923 | 40.63-58.99 |
| | Dec-20 | 682 | 0-0 | 0.00-0.00 | 561-833 | 37.03-54.98 |
| | Jan-21 | 597 | 0-0 | 0.00-0.00 | 621-891 | 41.74-59.87 |
| | Feb-21 | 602 | 0-0 | 0.00-0.00 | 597-868 | 40.62-59.04 |
| | Mar-21 | 682 | 0-0 | 0.00-0.00 | 539-786 | 36.72-53.54 |
| | Apr-21 | 607 | 0-0 | 0.00-0.00 | 566-801 | 40.19-56.90 |
| | May-21 | 540 | 0-0 | 0.00-0.00 | 575-799 | 42.94-59.68 |
| | Jun-21 | 563 | 0-0 | 0.00-0.00 | 518-726 | 40.20-56.31 |
| | Jul-21 | 515 | 0-0 | 0.00-0.00 | 553-749 | 43.75-59.26 |
| | Aug-21 | 525 | 0-0 | 0.00-0.00 | 494-690 | 40.67-56.78 |
| | Sep-21 | 512 | 0-0 | 0.00-0.00 | 492-665 | 41.81-56.49 |
| | Oct-21 | 378 | 0-0 | 0.00-0.00 | 588-758 | 51.77-66.72 |
| Tuberculosis care beds | | | | | | |
| | Jun-20 | 856 | 158-74 | 22.62-10.60 | 0-0 | 0.00-0.00 |
| | Jul-20 | 1198 | 489-412 | 68.86-58.07 | 0-0 | 0.00-0.00 |
| | Aug-20 | 959 | 271-190 | 39.31-27.60 | 0-0 | 0.00-0.00 |

| | | | | | | |
|---|---|---|---|---|---|---|
| | Sep-20 | 696 | 10-0 | 1.51-0.00 | 0-0 | 0.00-0.00 |
| | Oct-20 | 719 | 55-0 | 8.32-0.00 | 0-0 | 0.00-0.00 |
| | Nov-20 | 728 | 60-0 | 8.99-0.00 | 0-0 | 0.00-0.00 |
| | Dec-20 | 849 | 204-123 | 31.65-19.07 | 0-0 | 0.00-0.00 |
| | Jan-21 | 760 | 127-52 | 20.03-8.21 | 0-0 | 0.00-0.00 |
| | Feb-21 | 549 | 0-0 | 0.00-0.00 | 1-74 | 0.16-11.93 |
| | Mar-21 | 750 | 116-41 | 18.38-6.47 | 0-0 | 0.00-0.00 |
| | Apr-21 | 767 | 126-55 | 19.61-8.58 | 0-0 | 0.00-0.00 |
| | May-21 | 774 | 139-64 | 21.97-10.08 | 0-0 | 0.00-0.00 |
| | Jun-21 | 651 | 9-0 | 1.47-0.00 | 0-0 | 0.00-0.00 |
| | Jul-21 | 800 | 142-68 | 21.52-10.33 | 0-0 | 0.00-0.00 |
| | Aug-21 | 990 | 352-274 | 55.12-42.93 | 0-0 | 0.00-0.00 |
| | Sep-21 | 770 | 136-57 | 21.54-9.00 | 0-0 | 0.00-0.00 |
| | Oct-21 | 613 | 0-0 | 0.00-0.00 | 0-1 | 0.00-0.17 |
| Psychiatric care beds | | | | | | |
| | Jun-20 | 32144 | 0-0 | 0.00-0.00 | 0-1319 | 0.00-3.94 |
| | Jul-20 | 32390 | 0-0 | 0.00-0.00 | 0-1173 | 0.00-3.50 |
| | Aug-20 | 30341 | 0-0 | 0.00-0.00 | 0-2382 | 0.00-7.28 |
| | Sep-20 | 30351 | 0-0 | 0.00-0.00 | 0-1857 | 0.00-5.77 |
| | Oct-20 | 32266 | 932-0 | 2.97-0.00 | 0-0 | 0.00-0.00 |
| | Nov-20 | 28421 | 0-0 | 0.00-0.00 | 724-3217 | 2.29-10.17 |
| | Dec-20 | 30362 | 0-0 | 0.00-0.00 | 0-644 | 0.00-2.08 |
| | Jan-21 | 26791 | 0-0 | 0.00-0.00 | 1989-3894 | 6.48-12.69 |
| | Feb-21 | 26582 | 0-0 | 0.00-0.00 | 1664-4112 | 5.42-13.40 |
| | Mar-21 | 32354 | 954-0 | 3.04-0.00 | 0-0 | 0.00-0.00 |
| | Apr-21 | 31299 | 0-0 | 0.00-0.00 | 0-1151 | 0.00-3.55 |
| | May-21 | 27405 | 0-0 | 0.00-0.00 | 2527-4966 | 7.81-15.34 |
| | Jun-21 | 31345 | 0-0 | 0.00-0.00 | 0-1898 | 0.00-5.71 |
| | Jul-21 | 31038 | 0-0 | 0.00-0.00 | 133-2146 | 0.40-6.47 |
| | Aug-21 | 29525 | 0-0 | 0.00-0.00 | 392-2889 | 1.21-8.91 |
| | Sep-21 | 29254 | 0-0 | 0.00-0.00 | 281-2591 | 0.88-8.14 |
| | Oct-21 | 30545 | 0-0 | 0.00-0.00 | 0-598 | 0.00-1.92 |
| Total | | | | | | |
| | Jun-20 | 1226593 | 0-0 | 0.00-0.00 | 83468-175615 | 5.95-12.52 |
| | Jul-20 | 1286794 | 0-0 | 0.00-0.00 | 62332-142381 | 4.36-9.96 |
| | Aug-20 | 1292224 | 0-0 | 0.00-0.00 | 27789-116920 | 1.97-8.30 |
| | Sep-20 | 1255682 | 0-0 | 0.00-0.00 | 59849-147242 | 4.27-10.50 |
| | Oct-20 | 1338960 | 0-0 | 0.00-0.00 | 0-41654 | 0.00-3.02 |
| | Nov-20 | 1268112 | 0-0 | 0.00-0.00 | 35215-123443 | 2.53-8.87 |
| | Dec-20 | 1267513 | 0-0 | 0.00-0.00 | 34334-121881 | 2.47-8.77 |
| | Jan-21 | 1236277 | 0-0 | 0.00-0.00 | 56035-137972 | 4.08-10.04 |
| | Feb-21 | 1118830 | 0-0 | 0.00-0.00 | 177416-267804 | 12.79-19.31 |
| | Mar-21 | 1336421 | 0-0 | 0.00-0.00 | 0-26201 | 0.00-1.92 |
| | Apr-21 | 1266674 | 0-0 | 0.00-0.00 | 36781-121305 | 2.65-8.74 |
| | May-21 | 1190820 | 0-0 | 0.00-0.00 | 100191-191439 | 7.25-13.85 |
| | Jun-21 | 1259108 | 0-0 | 0.00-0.00 | 68302-157973 | 4.82-11.15 |
| | Jul-21 | 1289926 | 0-0 | 0.00-0.00 | 68879-153906 | 4.77-10.66 |
| | Aug-21 | 1340790 | 0-0 | 0.00-0.00 | 0-82950 | 0.00-5.83 |

| | | | | | | |
|---|---|---|---|---|---|---|
| | Sep-21 | 1226876 | 0-0 | 0.00-0.00 | 94591-185212 | 6.70-13.12 |
| | Oct-21 | 1286355 | 0-0 | 0.00-0.00 | 23532-108962 | 1.69-7.81 |
| Long-term care beds | | | | | | |
| | Jun-20 | 34390 | 0-0 | 0.00-0.00 | 2105-5122 | 5.33-12.96 |
| | Jul-20 | 34774 | 0-0 | 0.00-0.00 | 2288-5189 | 5.73-12.99 |
| | Aug-20 | 34214 | 0-0 | 0.00-0.00 | 2138-5073 | 5.44-12.91 |
| | Sep-20 | 34875 | 0-0 | 0.00-0.00 | 1731-4707 | 4.37-11.89 |
| | Oct-20 | 37202 | 0-0 | 0.00-0.00 | 0-2202 | 0.00-5.59 |
| | Nov-20 | 34245 | 0-0 | 0.00-0.00 | 3803-6757 | 9.28-16.48 |
| | Dec-20 | 38609 | 0-0 | 0.00-0.00 | 0-2401 | 0.00-5.85 |
| | Jan-21 | 33895 | 0-0 | 0.00-0.00 | 4264-7216 | 10.37-17.55 |
| | Feb-21 | 33363 | 0-0 | 0.00-0.00 | 4468-7366 | 10.97-18.08 |
| | Mar-21 | 38780 | 0-0 | 0.00-0.00 | 0-2166 | 0.00-5.29 |
| | Apr-21 | 36527 | 0-0 | 0.00-0.00 | 1587-4500 | 3.87-10.97 |
| | May-21 | 31525 | 0-0 | 0.00-0.00 | 5628-8448 | 14.08-21.13 |
| | Jun-21 | 35823 | 0-0 | 0.00-0.00 | 1286-4146 | 3.22-10.37 |
| | Jul-21 | 34744 | 0-0 | 0.00-0.00 | 2736-5551 | 6.79-13.78 |
| | Aug-21 | 36198 | 0-0 | 0.00-0.00 | 538-3369 | 1.36-8.51 |
| | Sep-21 | 34860 | 0-0 | 0.00-0.00 | 1916-4748 | 4.84-11.99 |
| | Oct-21 | 36284 | 0-0 | 0.00-0.00 | 554-3375 | 1.40-8.51 |
